# Shortening and Bending in Fractures of the Femur

**Published:** 1873-05

**Authors:** J. E. Nichols

**Affiliations:** Osage, Iowa


### Article III.—*Shortening and Bending in Fractures of the Femur.*

By J. E. Nichols, M.D., of Osage, Iowa.

Dr. M. A. McClelland makes a quotation in his article on "*Civil Malpractice*," appearing in the April No. of the Journal, which is deeply interesting to me. It is the strictures of Dr. Hamilton upon those surgeons who claim to have cured fractures of the femur without shortening. That so many eminent names should be ranged upon opposite sides of apparently so simple a question, is startling at first thought, yet a little reflection induces us to conclude that both parties are right and truthful. If a fractured arm may be so carefully cared for, and securely dressed, as to obtain a perfect limb, with no perceptible shortening or bending, it seems as though a leg might occasionally afford equally as happy a result. It is true that the latter is possessed of a larger amount of muscular tissue; but this is hardly sufficient to make all the difference, and does not.

From the limited observations I have been permitted to make, both in army and private practice, I am convinced that the necessity for lying in bed during the treatment of a fractured femur, and the temptation to use the limb too soon afterwards, are the chief causes of all the deformities. I have not had the facilities for observing variety in these cases that have been enjoyed by many surgeons; but I have had one experience that falls to the lot of few, and is desired by none. It was a fractured thigh occurring to myself.

This case, with an account of a fracture-couch that I invented at the time, was reported in the CHICAGO MEDICAL JOURNAL for December, 1860, if I rightly remember. My preceptor, Dr. O. D. Howell, had taken every pains to secure a perfect limb. I was a student of medicine at the time, and he placed before me every volume in his library that treated of fractures for my study, urged me to lie perfectly still, and insisted that my parents and attendants should keep me wholly immovable while attending to my wants. Besides this, he came to see me once a day to look after extension and counter-extension himself. The result of this zealous care was apparent on removing the splints at the end of seven weeks. No shortening could be found by the most careful measurement. In measuring to the malleolus the whole limb was found to be longer than the other by a fourth of an inch, caused, I have no doubt, by the extension force drawing apart the bones at the knee joint. A starch bandage being applied, I soon began to walk with crutches, finally using only a cane, and as soon as possible I threw that to one side.

It was about eighteen months afterwards, that I reported the case in the JOURNAL, and shortly thereafter Prof. Brainard called me into his office one day as I was passing, and made a thorough examination of the injured thigh. On measuring it, he found, to my great discomfiture, a full half-inch of shortening, and a slight bending backwards of the point of fracture. Several persons of proved mechanical skill had been present at the time the dressing was removed by Dr. Howell, and all of them, after the closest scrutiny, pronounced it a perfect limb. More attention was paid to the matter than would otherwise have been, on account of one of our surgeons recently having had a suit for malpractice to defend.

Up to the time Dr. Brainard made the examination I had not the least thought that one of my limbs was shorter than the other. From that time until A. D. 1865, it continued to shorten still more; for when Prof. Freer examined my physical status, at this latter date, he not only discovered a lateral curvature of the spine but a shortening of the thigh to the extent of *three-fourths of an inch.* From that date to the present time no further shortening has occurred, as I have just had verified by actual measurement.

Now, with regard to those surgeons who report cures of fractures of the femur without shortening, were not their measurements and records made at the close of their most careful course of treatment, when all they reported was true and correct? While there has been some shortening in nearly all the cases coming under my observation *during treatment*, there has been infinitely more shortening and especially bending after all dressings were removed in the few cases I have been able to keep a watch upon; and I presume the same is true of the others.

To keep a patient off his leg until it becomes so firm that it will not shorten further, would be a confinement that few or none would consent to. During the treatment there is only the force which is exerted by the muscles to overcome, and this, when they are in a state of repose (unexcited) is not so great as many would suppose. The muscles at the instant of applying extension, obstinately resist a great force; but keep up the extension, and as they gradually yield themselves to relaxation, the power of extension is found to be much too great for all that is required of it. After treatment is discontinued, when the limb is first employed, there is not only the muscular force to overcome, but the weight of the body to sustain besides.

A person with a fractured arm will carry it about suspended in complete repose long after he might use it with comparative safety; and when he does use it, the exertion of force is in a direction to prevent, more than to produce, shortening, while the weight of the arm below the fracture offers its gravity to antagonize the muscular force. An arm is more liable to become crooked by premature use after fracture than shortened. But it is an easy matter for most patients to deny themselves the use of an arm until it may be safely employed, while a leg is tested and put into use just as soon as it will bear it without visibly yielding. Why, if a man sprains his wrist he will carry it around for days or even weeks; but if the ankle is so unfortunate as to become sprained, it will be put to the task of limping down town the very next day and perhaps is walked home on immediately after the injury.

There is no comparison between the two extremities. The attendant conditions of the arm and leg, from the moment of fracture until complete ossification of the provisional callus occurs, are entirely and widely different. The broken arm is taken up tenderly, and cleverly carried by the patient himself, often to the surgeon's office; and from that time until it is cured he acquires a habit of protecting it from the smallest shock even in his sleep; while a broken leg makes of a man a helpless, dead weight, to be moved about and cared for by others. There is a vast difference in the muscular antagonism of a fractured limb when handled by the patient himself and when manipulated by others.

Surgeons cannot be too particular about a bed for their patient with a fractured femur. Such a patient is supposed to have no knowledge of all he must experience and endure in being confined to his bed, and of the necessity of his being patient and immovable, however much his sturdy back and hardy muscles may ache from inaction. But the surgeon is presumed to understand and fully appreciate this; and ought not only to indifferently desire a properly constructed bed, but should insist that one be obtained or improvised for the occasion. He ought not only to advise, but should repeat and reiterate it, that his patient must lie still. When he removes the dressings he should inform his patient that it all depends upon himself as to whether he shall have as good a limb six months hence as now, or a shorter and more crooked one. All these things should be forcibly impressed upon the mind of the patient. The duller the mind the greater need of careful explanations, and of thoroughly "beating in" the instructions. I have often thought that some appliance to aid in walking, on first making use of a fractured femur, as in hip-joint disease, would save many limbs from serious deformity.

